# Impact of Apheresis on Lithium Levels in a Patient with Multiple Myeloma and Bipolar Disorder: A case report

**DOI:** 10.1192/j.eurpsy.2025.1093

**Published:** 2025-08-26

**Authors:** D. Üzüm, M. R. Altunel, N. G. Usta Sağlam, S. Turan

**Affiliations:** 1 Department of Psychiatry, Istanbul University - Cerrahpasa, Cerrahpasa Medical School, Istanbul, Türkiye

## Abstract

**Introduction:**

Managing lithium levels in patients with coexisting medical conditions can present several challenges. Here, we report a case of a bipolar disorder (BD) patient on lithium therapy who underwent apheresis in the hematology department for autologous stem cell transplantation preparation.

**Objectives:**

Bipolar disorder and multiple myeloma are both challenging conditions to manage individually, but their combination presents unique difficulties. This report details the case of a patient with both conditions, emphasizing the impact of a major medical intervention on psychiatric health.

**Methods:**

Presentation of a patient’s case regarding the impact of apheresis on lithium levels.

**Results:**

A 52-year-old woman presented in January 2024 with bilateral hip pain, and MRI revealed lesions on the T12 vertebra, which were identified as multiple myeloma on a PET scan. She underwent neurosurgical intervention for the spinal lesions and was planned for autologous stem cell transplantation by the hematology team, involving apheresis and filgrastim administration. The patient has a 20-year history of BD and and has never been hospitalized in a psychiatric facility. Her condition has remained stable with lithium and quetiapine, especially when lithium levels were maintained between 0.8-0.9 mEq/L. During acute episodes, increasing the quetiapine dosage and adding clonazepam have proven effective. It was noted that her most recent hypomanic episode occured in January 2024, due to the interruption of her medications during her admission to the neurosurgery ward. When the patient was admitted to the hematology ward for apheresis, she was taking 1200 mg/day of lithium and 600 mg/day of quetiapine, with a blood lithium level of 0.56 mmol/L. Since her blood lithium levels were considered low, the dosage was increased to 1500 mg/day. One week later, the patient developed complaints of increased amount of speech, overspending and irritability. She was evaluated in consultation and hypomania was considered as a result of psychiatric examination. Her blood lithium level had decreased to 0.46 mmol/L at that time. Clonazepam 0.5 mg/daily was added to her treatment. The apheresis treatment was completed after 10 days. Four days after the completion of the apheresis therapy, her lithium level increased to 0.81 mmol/L and her hypomanic symptoms have improved.

**Conclusions:**

Although lithium is well-documented to be effectively removed via hemodialysis and peritoneal dialysis, its removal through apheresis is not documented. Managing BD in patients undergoing apheresis presents challenges due to its impact on plasma lithium levels. This case underscores the importance of individualized treatment strategies, including frequent monitoring of serum lithium levels and timely dose adjustments. Clinicians must remain cautious in patients undergoing plasma exchange to maintain mood stability and adjust treatments as necessary.

**Disclosure of Interest:**

None Declared

